# Biomarkers of Leucine‐Rich Repeat Kinase 2 (LRRK2) and Lysosomal Dysfunction in Progressive Supranuclear Palsy

**DOI:** 10.1002/mds.70295

**Published:** 2026-04-15

**Authors:** Louise‐Kristine Nielsen, Joshua L.I. Frost, David P. Vaughan, Raquel Real, Riona Fumi, Marte Theilmann Jensen, Megan Hodgson, Eleanor J. Stafford, Lesley Wu, Olaf Ansorge, Annelies Quaegebeur, Kieren S.J. Allinson, Thomas T. Warner, Zane Jaunmuktane, Anjum Misbahuddin, P. Nigel Leigh, Boyd C.P. Ghosh, Kailash P. Bhatia, Alistair Church, Christopher Kobylecki, Michele T.M. Hu, James B. Rowe, Alan A. Shomo, Danielle L. Graham, Omar S. Mabrouk, Huw R. Morris, Esther M. Sammler, Edwin Jabbari

**Affiliations:** ^1^ Medical Research Council Protein Phosphorylation and Ubiquitylation Unit University of Dundee Dundee UK; ^2^ Department of Clinical and Movement Neurosciences UCL Queen Square Institute of Neurology London UK; ^3^ Movement Disorders Centre UCL Queen Square Institute of Neurology London UK; ^4^ Nuffield Department of Clinical Neurosciences University of Oxford Oxford UK; ^5^ Department of Histopathology Cambridge University Hospitals NHS Foundation Trust Cambridge UK; ^6^ Department of Imaging and Pathology University of Leuven Leuven Belgium; ^7^ Reta Lila Weston Institute UCL Queen Square Institute of Neurology London UK; ^8^ Queen Square Brain Bank for Neurological Disorders UCL Queen Square Institute of Neurology London UK; ^9^ Department of Neurology Queen's Hospital Romford UK; ^10^ Department of Neuroscience, Brighton and Sussex Medical School University of Brighton and University of Sussex Brighton UK; ^11^ Wessex Neurological Centre University Hospitals Southampton NHS Foundation Trust Southampton UK; ^12^ Department of Neurology Royal Gwent Hospital Newport UK; ^13^ Department of Neurology, Northern Care Alliance NHS Foundation Trust, Manchester Academic Health Science Centre University of Manchester Manchester UK; ^14^ Department of Clinical Neurosciences, Cambridge University Hospitals NHS Trust and MRC Cognition and Brain Sciences Unit University of Cambridge Cambridge UK; ^15^ Neurodegenerative Diseases Research Unit, Biogen Cambridge Massachusetts USA

## Abstract

**Background:**

Common and rare genetic variants in leucine‐rich repeat kinase 2 (LRRK2) have been linked with sporadic and familial Parkinson's disease (PD). Recently, we discovered that common genetic variation near the LRRK2 locus determined survival in progressive supranuclear palsy (PSP). Our study aimed to explore biomarkers of LRRK2 and lysosomal dysfunction in PSP.

**Methods:**

Immunoblotting was used to measure total LRRK2 and LRRK2‐dependent Rab10 phosphorylation at the threonine 73 residue (pRab10^Thr73^) in neutrophil and monocyte samples from PSP and control participants. Urine samples were applied to a multiplexed assay to quantitate bis(monoacylglycerol)phosphate (BMP) species as markers of lysosomal dysfunction. Cerebrospinal fluid (CSF) samples from a wider cohort of PSP and control participants were applied to a stable isotope standards and capture by anti‐peptide antibodies assay to measure total LRRK2 and pRab10^Thr73^ levels. LRRK2 genotypes (rs76904798 and rs2242367) and 1‐year change in Progressive Supranuclear Palsy Rating Scale (PSPRS) scores were obtained.

**Results:**

A total of 61 PSP and 34 control participants were included. Total urine 22:6‐BMP levels were higher in PSP versus control samples (*P* = 0.04) and correlated with CSF total LRRK2 levels (*r* = 0.49, *P* = 0.04). There were no group‐level differences in monocyte and CSF levels of total LRRK2 and pRab10^Thr73^. In PSP, carriers of the alternate allele (CT and TT genotypes) at the LRRK2 PD risk variant, rs76904798, had higher levels of CSF total LRRK2 versus CC genotype (*P* = 0.02). Baseline monocyte total LRRK2 levels predicted 1‐year change in the PSPRS score (*P* = 0.008).

**Conclusions:**

Biochemically defined lysosomal dysfunction is evident in PSP. Genetic and biochemical stratification may identify PSP patients that would benefit from LRRK2‐targeting therapies. © 2026 The Author(s). *Movement Disorders* published by Wiley Periodicals LLC on behalf of International Parkinson and Movement Disorder Society.

## Introduction

Progressive supranuclear palsy (PSP) and Parkinson's disease (PD) are neurodegenerative parkinsonian disorders with no effective disease‐modifying therapies. Although they have overlapping clinical features, the two disorders are pathologically distinct, with PD being characterized by the presence of alpha‐synuclein‐related neuronal Lewy body pathology and PSP being characterized by neuronal and astrocytic four‐repeat tau (4RT) pathology.^1^

Around 1% of all PD and 5% of familial PD cases are due to rare pathogenic mutations in the leucine‐rich repeat kinase 2 (LRRK2) gene (LRRK2‐PD), with the G2019S variant being the most common cause of familial PD in populations of European ancestry.^2^ Additionally, common genetic variation at LRRK2 (rs76904798) is a well‐established risk locus for sporadic PD.^3^ LRRK2 mutations have rarely been associated with PSP, with pathogenic LRRK2 variants identified in 0.3% of a large postmortem PSP cohort.^4^ However, we have recently shown that common variation near the LRRK2 locus (rs2242367) is a genetic determinant of survival in a genome‐wide association study of over 1000 pathologically diagnosed PSP participants.^5^

Interestingly, the neuropathology of LRRK2‐PD is unclear, as typical Lewy body pathology is absent in around half of all cases that come to postmortem, with previous studies highlighting the common presence of tau pathology.^6^ This is also reflected in alpha‐synuclein seed amplification (a‐syn SAA) studies of PD, where around 30–40% of LRRK2‐PD participants are α‐syn SAA‐negative.^7^

LRRK2 encodes a multidomain protein with catalytic GTPase and kinase domains. In PD, pathogenic LRRK2 variants cluster in these two catalytic domains and increase LRRK2 kinase activity with subsequent hyperphosphorylation of its endogenous targets, a subgroup of RabGTPases. The resulting activation leads to pathogenic downstream effects, including impaired vesicle trafficking, lysosomal dysfunction, and neuroinflammation related to microglial activation, all of which may contribute to PD pathogenesis.^8^ As such, LRRK2 kinase inhibitors and LRRK2‐lowering therapies, including antisense oligonucleotides (ASO), are currently being trialled in both sporadic PD and LRRK2‐PD.^9^

Translational assays have been developed to probe LRRK2 function in peripheral blood neutrophils and monocytes where LRRK2 is highly expressed. Quantitative immunoblotting and more sensitive mass spectrometry assays have enabled the quantification of total and phosphorylated levels of both LRRK2 and a subgroup of LRRK2 phosphorylated RabGTPases, including Rab10 at the threonine 73 residue (pRab10^Thr73^), in neutrophil and monocyte samples as markers of LRRK2 kinase activity.^10^ Previous studies have shown significantly higher neutrophil and monocyte levels of Rab10 phosphorylation in LRRK2‐PD (R1441G mutation) versus controls, a non‐significant trend towards similar results in LRRK2‐PD (G2019S mutation) versus controls, and no difference in levels between sporadic PD and controls.^11^ Similarly, urine levels of bis(monoacylglycerol)phosphate (BMP) species, a marker of lysosomal dysfunction, have been shown to be raised in both manifesting and non‐manifesting LRRK2‐PD versus sporadic PD, whereas there is no significant difference in levels between sporadic PD and controls.^12^ Furthermore, in the Parkinson's Progression Markers Initiative (PPMI) cohort, urine BMP levels were shown to be elevated in PD participants who were carriers of the GBA1 N409S risk variant versus controls, but this was much smaller than the three‐to‐seven‐fold elevation seen in LRRK2‐PD versus controls.^13^

In this study, we explored whether blood and cerebrospinal fluid (CSF) biomarkers of the LRRK2 pathway and urine biomarkers of lysosomal dysfunction are: (1) deranged in PSP versus controls; (2) determined by LRRK2 single nucleotide polymorphisms (protein quantitative trait loci [pQTLs]); and (3) predictive of clinical disease progression in PSP.

## Patients and Methods

### Patient Consent and Cohorts

All participants gave written informed consent for the use of their medical records and biosamples for research purposes, including the analysis of DNA. Participants from the PROSPECT‐UK study (Queen Square Research Ethics Committee 14/LO/1575) who fulfilled at least “possible” clinical diagnostic criteria for any PSP phenotype were included in this study.^14^, ^15^ Age‐ and sex‐matched neurologically normal controls from the PROSPECT‐UK study were also included. Postmortem evaluation was conducted on a subset of participants with a clinical diagnosis of PSP.

### Clinical Data Collection

The following clinical variables and measures were collected from PSP participants: sex; age at symptom onset; age and disease duration at baseline assessment; baseline and 1‐year follow‐up scores for the PSP Rating Scale (PSPRS), Movement Disorder Society‐Unified Parkinson's Disease Rating Scale‐Part III (MDS‐UPDRS‐III) in the off state, and Montreal Cognitive Assessment (MoCA).

### Neutrophil and Monocyte LRRK2 and pRab10 Detection by Quantitative Immunoblotting

For each participant, neutrophil and monocyte samples were isolated from fresh whole blood samples, divided into two and treated with either DMSO (inactive) or MLi‐2 (LRRK2 inhibitor), and stored at −70°C within 3 hr of blood sampling as previously described.^10^ Multiplexed immunoblotting was carried out using stored neutrophil and monocyte samples as per established protocols (Supplementary Methods and Table S1). The quantified signal intensities of the total protein bands were normalized to those of the housekeeping protein (total LRRK2/GAPDH and total Rab10/GAPDH), whereas the phosphorylated species were normalized to the total protein signal intensities (pSer935/total LRRK2 and pThr73/total Rab10). All measurements were then normalized against an intergel control, which was run on all gels to control for variability.

### CSF LRRK2 and pRab10 Detection by SISCAPA

A subset of the PSP and control participants that had undergone fresh blood and urine sampling also had a lumbar puncture to obtain CSF for LRRK2 and pRab10 testing. Importantly, these CSF assays are able to be performed on samples stored at −70°C with no preprocessing required and so CSF samples previously obtained from additional PSP and control PROSPECT‐UK participants were also included. However, we only used CSF samples that had not previously undergone freeze–thaw cycles.

CSF aliquots (500 μL) were spiked with 1X RIPA (Millipore Sigma 20–188). Samples were then spiked with 1 pg of LRRK2 stable isotope‐labeled peptide K_13C(6)15N (2)_ AEEGDLLVNPDQPR_13C(6)15N (2)_ and 100 fg of pRab10 Thr73 stable isotope‐labeled peptide FHTITTSYYR_13C(6)15N (2)_ as internal standards. Samples were then digested for 1.5 hr at 37°C with 10 μg TPCK‐treated trypsin (Millipore Sigma T1426). N241A/34 LRRK2 antibody (Antibodies Inc. 75‐253) and MJF‐R20 pRab8A antibody (Abcam ab230260) were biotinylated then immobilized on AssayMAP 5 μL streptavidin SA‐W cartridges (Agilent Technologies G5496‐60010) at a ratio of 1 μg of each antibody per cartridge. Digested samples were then loaded onto the AssayMAP Bravo for peptide immunocapture. Eluted samples were loaded onto an Evosep One LC system running the 40SPD Whisper method coupled to a Thermo EASY‐Spray PepMap RSLC C18 column (Thermo Scientific ES904). Mass spectrometry was performed on a Thermo Exploris 480 operating in parallel reaction monitoring (PRM) mode. Targets included *m/z* 560.9566 [M + 3H]^3+^ (native LRRK2 peptide), *m/z* 566.9641 [M + 3H]^3+^ (LRRK2 internal standard), *m/z* 456.8710 [M + 3H]^3+^ (native pRab10 Thr73 phosphopeptide), and *m/z* 460.2071 [M + 3H]^3+^ (pRab10 Thr73 internal standard). PRM scans were collected at a resolution of 120,000 with AGC target set to standard (1 × 10^6^) and maximum injection time set to automatic detection. Higher‐energy collisional dissociation (HCD) was optimized at 20% and 25% for the LRRK2 and pRab10 peptide pairs, respectively. Quantitation and data analysis were performed in SkyLine 24.1.0.199.

### Urine BMP Detection

For each participant, fresh urine samples were obtained and centrifuged for 15 min at 2500 × g and 4°C before 1 mL of supernatant was stored at −70°C within 30 min of urine sampling. Stored urine samples were analyzed at Nextcea Inc. (Woburn, MA, USA) using a multiplexed ultra‐performance liquid chromatography tandem mass spectrometry (UPLC‐MS/MS) method to simultaneously quantitate BMP species (total di‐22:6‐BMP, 2,2′ di‐22:6‐BMP, 2,3′ di‐22:6‐BMP, 3,3′ di‐22:6‐BMP, total di‐18:1‐BMP, 2,2′ di‐18:1‐BMP, 2,3′ di‐18:1‐BMP, 3,3′ di‐18:1‐BMP) as per established protocols.^12^ Measured concentrations of urine BMPs (ng/mL) were divided by the concentration of urine creatinine and reported as ng/mg creatinine.

### Genetic Testing

DNA samples from PSP and control participants underwent polymerase chain reaction (PCR)‐based KASP genotyping at LGC Biosearch Technologies for the LRRK2‐G2019S variant (rs34637584), the LRRK2 PD risk variant (rs76904798), and the LRRK2 PSP survival variant (rs2242367). Whole‐genome sequencing (WGS) and Illumina Neurobooster Array (NBA)^16^ genotyping was performed on a subset of DNA samples that had initially undergone KASP genotyping at LGC Biosearch Technologies. WGS data were available via release 10 of the Global Parkinson's Genetics Program (https://gp2.org). We screened for variants defined as pathogenic, likely pathogenic, or risk factor according to ClinVar (https://www.ncbi.nlm.nih.gov/clinvar/) across genes known to be associated with PD/parkinsonism (Table S2). NBA data were screened for pathogenic variants across the same list of genes known to be associated with PD/parkinsonism as in the WGS data analysis (Table S2).

### Statistical Analyses

All statistical analyses were carried out using R version 4.5.1.

Urine BMP data and LRRK2 and Rab10 data from neutrophils, monocytes, and CSF were screened for extreme outliers, which were defined as values above the third quartile plus three times the interquartile range (IQR) or values below the first quartile minus three times the IQR. All results were individually checked for exclusion against published ranges for each biomarker.

Multinomial logistic regression via the nnet package was used to assess the association between group (PSP vs. controls) and neutrophil, monocyte, urine, and CSF biomarkers, adjusting for sex and age at testing. Linear regression analyses were used for pQTL analyses where neutrophil, monocyte, and CSF biomarker levels were stratified by LRRK2 PD risk (rs76904798, C:C vs. T:T/T:C) and PSP survival (rs2242367, G:G vs. A:A/A:G) single nucleotide polymorphism (SNP) status within each group separately – adjusting for sex, age, and disease duration at testing in the PSP group, and sex and age at testing in the control group. All the above analyses were visualized using violin plots from the ggplot2 package.

Assessment of correlation between neutrophil, monocyte, urine, and CSF biomarkers in the PSP and control groups was done using Spearman correlation with pairwise complete observations, visualized on heat maps. Statistically significant correlations were modeled linearly and adjusted for covariates.

In the PSP group, linear regression was used to model whether baseline levels of (a) neutrophil, monocyte, and CSF total LRRK2 and pRab10 and (b) urine total di‐22:6‐BMP and total di‐18:1‐BMP predicted 1‐year change in the PSP rating scale score as a primary outcome measure, adjusting for sex, age, and disease duration at baseline. We also carried out exploratory progression analyses using the same linear regression models to assess the relationship between baseline levels of our blood, urine, and CSF biomarkers versus 1‐year change in MDS‐UPDRS‐III and MoCA scale scores as secondary outcome measures. Significant relationships were visualized as scatter plots with linear fit lines.

Statistical significance was defined as a *P*‐value<0.05 for all analyses aside from the primary progression analysis where the threshold for *P*‐value significance was set using the Benjamini–Hochberg correction method^17^ for multiple testing with a false discovery rate of 5%.

## Results

A total of 61 PSP participants and 34 age‐ and sex‐matched controls from the PROSPECT‐UK study were included in this study. All six PSP participants that subsequently underwent postmortem analysis were found to have underlying 4RT neuropathology (five PSP, one corticobasal degeneration [CBD]) (Table 1). LGC genotyping was available for 50/61 (82%) PSP and 26/34 (76%) control participants. WGS data were available for 23/61 (38%) PSP and 5/34 (15%) control participants. No pathogenic variants were identified in PSP or control participants via WGS data. NBA data were available for an additional 18 PSP and 14 control participants that had not undergone WGS. This did not reveal any pathogenic variants.

**TABLE 1:** Clinical profile of the leucine‐rich repeat kinase 2 (LRRK2) biomarker cohort

Variable	PSP (N = 61)	Controls (N = 34)
Sex (% male)	56	38
Clinical subtype at baseline assessment (n)	PSP‐RS (37) PSP‐P (12) PSP‐PGF (4) PSP‐CBS (3) PSP‐F (3) PSP‐SL (2)	
Mean age at symptom onset, years (SD)*	64.8 (7.1)	
Mean age at baseline assessment, years (SD)*	68.7 (6.9)^†^	61.8 (12.5)
Mean disease duration at baseline assessment, years (SD)*	3.9 (2.0)	
Mean PSPRS at baseline assessment, score (SD)^	32.7 (12.4)	
Mean MDS‐UPDRS‐III at baseline assessment, score (SD)^	34.3 (13.6)	
Mean MoCA at baseline assessment, score (SD)^	21.8 (4.5)^†^	27.6 (1.6)
Participants deceased at censoring (n), mean disease duration from symptom onset to death, years (SD)	32 6.9 (2.8)	
Primary pathological diagnosis in participants undergoing postmortem (n)	PSP (5) CBD (1)	

*Note*: Fisher's exact test used for group comparisons of sex distributions. Group comparisons of continuous variables were done using linear regression that was either unadjusted (*) or adjusted (^) for sex, age, and disease duration at baseline (group comparisons of MoCA scores involving controls were only adjusted for sex and age at baseline). ^†^ *P* < 0.05 versus control group.

Abbreviations: PSP, progressive supranuclear palsy; PSP‐RS, PSP‐Richardson syndrome; PSP‐P, PSP‐parkinsonism; PSP‐PGF, PSP‐progressive gait freezing; PSP‐CBS, PSP/corticobasal syndrome; PSP‐F, PSP‐frontal; PSP‐SL, PSP‐speech and language disorder; SD, standard deviation; PSPRS, PSP Rating Scale; MDS‐UPDRS‐III, Movement Disorder Society‐Unified Parkinson's Disease Rating Scale‐Part III; MoCA, Montreal Cognitive Assessment; CBD, corticobasal degeneration.

**FIG. 1:**
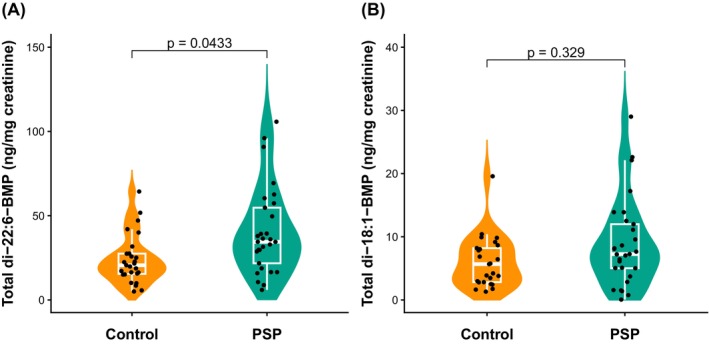
Urine bis(monoacylglycerol)phosphate (BMP) levels in progressive supranuclear palsy (PSP) and control participants. (A) Total di‐22:6 BMP levels. (B) Total di‐18:1‐BMP levels. Group comparisons were done using logistic regression that adjusted for sex and age at testing. [Color figure can be viewed at wileyonlinelibrary.com]

Fresh blood samples from 30 PSP and 30 control participants underwent neutrophil and monocyte extraction followed by treatment with DMSO and MLi‐2 before being stored at −70°C. Quantitative immunoblotting was subsequently performed on stored neutrophil and monocyte samples, and representative immunoblots are shown in Figure S1. Two PSP monocyte samples were excluded from analyses as it was not possible to accurately quantify their immunoblotting bands, which led to extreme outlier results. For the remaining neutrophil and monocyte samples, MLi‐2‐treated samples showed a depletion in the levels of pLRRK2^Ser935^ pRab10^Thr73^, which confirmed that the levels of LRRK2 and Rab10 phosphorylation in DMSO‐treated samples were mediated by LRRK2 kinase activity. We found no association between group (PSP vs. controls) and the levels of total and phosphorylated LRRK2 and Rab10 in DMSO‐treated monocyte samples. Similarly, there were no associations between group (PSP vs. controls) and the same measures in DMSO‐treated neutrophil samples except pRab10, which was lower in PSP versus controls (*P* = 0.03) (Fig. S2). From the same set of participants, 29/30 PSP and 28/30 control participants provided urine samples which underwent processing before being stored at −70°C. One PSP patient was unable to provide a urine sample due to advanced disease and urinary incontinence, and urine samples from two controls were excluded due to improper processing prior to storage. BMP assay testing was performed on stored urine samples. One control sample was excluded from analyses due to the presence of extreme outliers in the BMP data. Group level analyses revealed significantly higher levels of total di‐22:6‐BMP as well as the 3,3′ di‐18:1‐BMP isoform in PSP versus controls (*P* < 0.05) (Fig. 1) (Table S3).

**FIG. 2:**
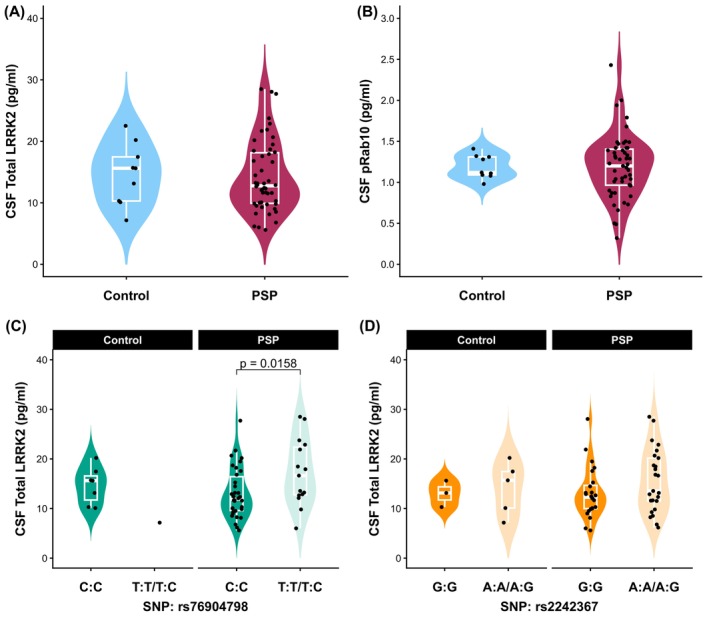
(A, B) Cerebrospinal fluid (CSF) total leucine‐rich repeat kinase 2 (LRRK2) and pRab10 levels in progressive supranuclear palsy (PSP) and control groups. Group comparisons were done using logistic regression that adjusted for sex and age at testing. (C, D) CSF total LRRK2 levels in PSP and control groups stratified by rs76904798 and rs2242367 genotype status. Genotype group comparisons were done using linear regression that adjusted for sex, age, and disease duration at testing (control group comparisons adjusted for sex and age at testing) with GG (rs2242367) and CC (rs76904798) as reference groups. SNP, single nucleotide polymorphism. [Color figure can be viewed at wileyonlinelibrary.com]

Stored CSF samples from 51 PSP and 9 control participants underwent testing for total LRRK2 and pRab10^Thr73^ levels. There was no association between group (PSP vs. controls) and CSF total LRRK2 and pRab10 levels (Fig. 2A,B). Using LGC genotype data, we then stratified our blood (neutrophil and monocyte) and CSF measures of total LRRK2 and pRab10 in each group separately by LRRK2 PD risk (rs76904798) and PSP survival (rs2242367) genotype status to look for state‐specific pQTLs. This analysis revealed that in PSP, carriers of the alternate ‘T’ allele (CT and TT genotypes) at rs76904798 had higher levels of CSF total LRRK2 versus CC genotype (*P* = 0.02) (Fig. 2C). A non‐significant (*P* = 0.08) trend towards rs2242367 genotype status influencing CSF total LRRK2 levels was observed in the PSP group (Fig. 2D). In controls, rs2242367 genotype status was associated with neutrophil total LRRK2 levels (*P* = 0.04). In the PSP group, there were no pQTL associations observed in (a) neutrophil and monocyte levels of total LRRK2 and pRab10 and (b) CSF levels of pRab10 (Table S4). Of note, in the 24 PSP and 8 control participants who had undergone both LGC genotyping and WGS, there was 100% concordance in the genotype results for rs76904798 and rs2242367.

**FIG. 3:**
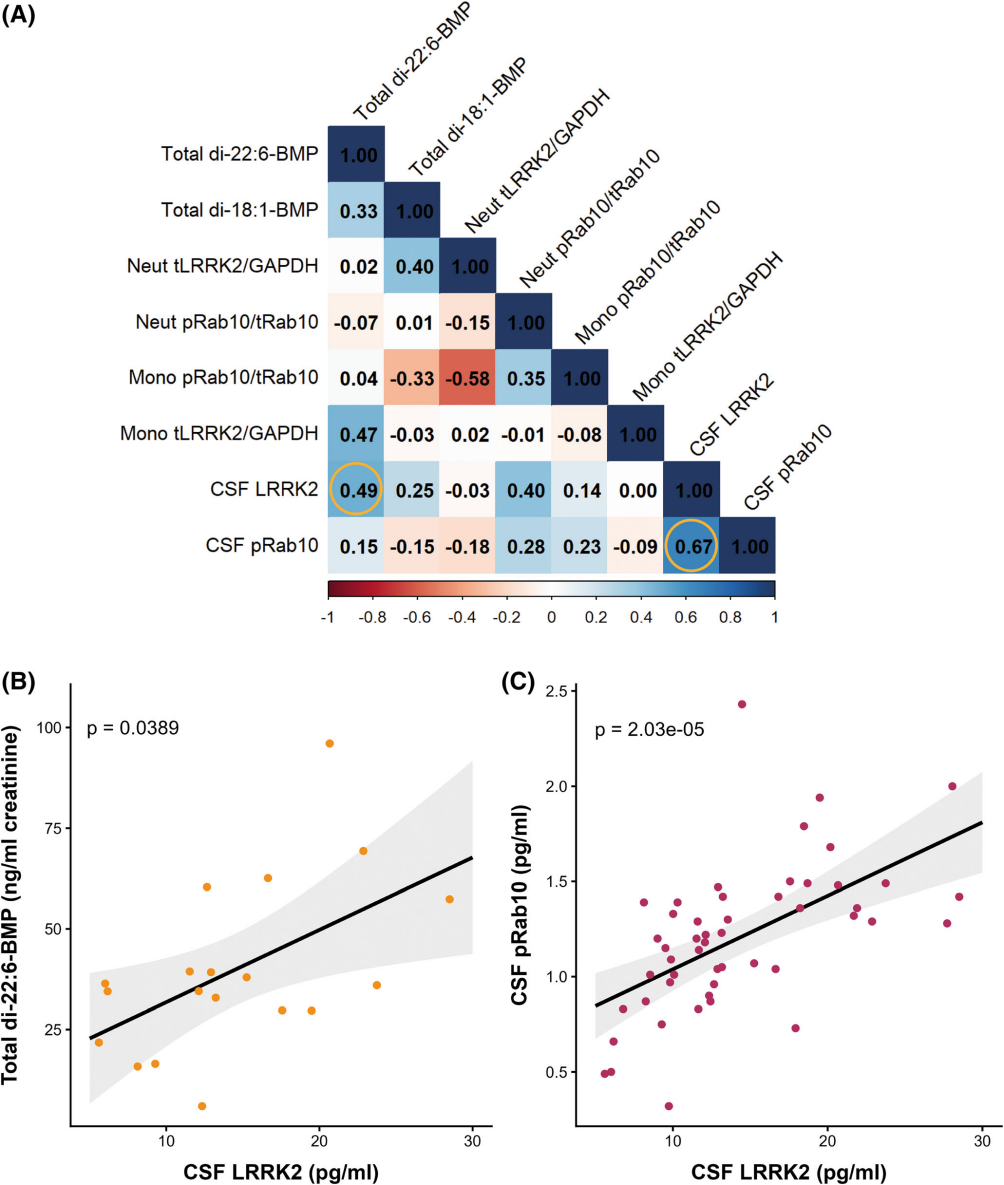
(A) Heatmap of Spearman's rho biomarker correlations in the progressive supranuclear palsy (PSP) group with *r* values highlighted. Circles represent correlations that were also significant in the linear regression analyses. (B, C) Urine total di‐22:6 bis(monoacylglycerol)phosphate (BMP) versus cerebrospinal fluid (CSF) leucine‐rich repeat kinase 2 (LRRK2) and CSF LRRK2 versus CSF pRab10 linear plots. Linear regression analyses that adjusted for sex, age, and disease duration at testing were used to generate *P*‐values. [Color figure can be viewed at wileyonlinelibrary.com]

We assessed the correlation between each of the blood, urine, and CSF biomarkers in PSP and control groups, respectively. Of note, this included 19 PSP and 3 control participants who had undergone testing for each of the blood, urine, and CSF measures. In the PSP group, the only correlations that were statistically significant in linear regression analyses that adjusted for sex, age, and disease duration at testing were urine total di‐22:6 BMP versus CSF LRRK2 (*r* = 0.49, *P* = 0.04) and CSF LRRK2 versus CSF pRab10 (*r* = 0.67, *P* < 0.0001) (Fig. 3).

Of note, there were no significant correlations between neutrophil or monocyte LRRK2 versus pRab10, neutrophil or monocyte LRRK2 versus CSF LRRK2, and neutrophil or monocyte pRab10 versus CSF pRab10. In the control group, the only correlation that was statistically significant in linear regression analyses that adjusted for sex and age at testing was neutrophil pRab10 versus monocyte pRab10 (Fig. S3).

**FIG. 4:**
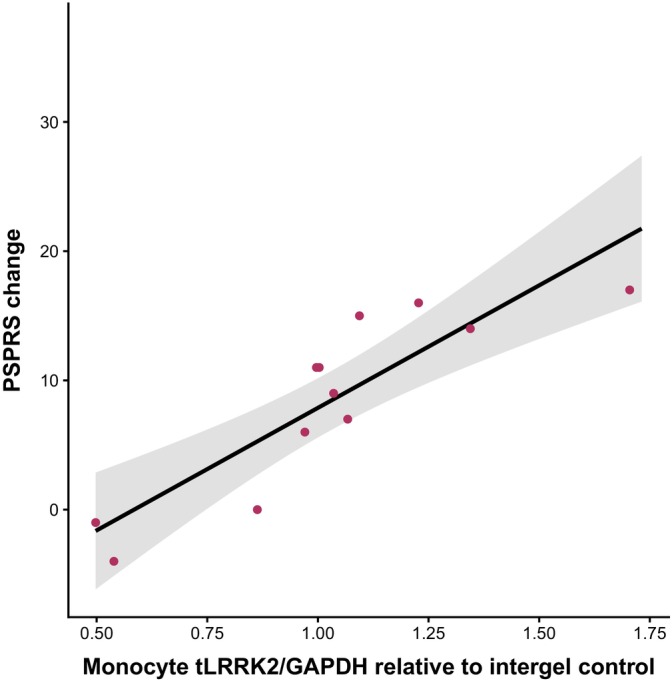
Baseline monocyte total leucine‐rich repeat kinase 2 (LRRK2) levels versus 1‐year change in the Progressive Supranuclear Palsy Rating Scale (PSPRS) score. [Color figure can be viewed at wileyonlinelibrary.com]

Finally, in the PSP group we assessed whether baseline levels of the blood, urine, and CSF biomarkers predicted 1‐year change in the PSPRS score as a primary outcome measure via linear regression models that adjusted for sex, age, and disease duration at baseline. Of note, 1‐year clinical PSPRS scores were available in 12/30 (40%) of participants that had undergone baseline blood and urine testing, and 28/51 (55%) of participants that had undergone baseline CSF testing. The average 1‐year change in the PSPRS score was 12.0 points, standard deviation 10.3. The progression analyses revealed that baseline monocyte total LRRK2 levels predicted 1‐year change in the PSPRS score (*P* = 0.008) (Fig. 4) (Table S5). In the exploratory analyses of secondary outcome measures, there were no significant associations between baseline levels of our blood, urine, and CSF biomarkers versus 1‐year change in MDS‐UPDRS‐III and MoCA scale scores (Table S6).

## Discussion

In this study we have comprehensively characterized the profile of fluid biomarkers of LRRK2 and lysosomal dysfunction in PSP and assessed their association with genetic variation at the LRRK2 locus and clinical disease progression.

First, we used an established quantitative immunoblotting assay and found no evidence of association between group (PSP vs. controls) and levels of monocyte total and phosphorylated LRRK2 and Rab10. Our finding of lower levels of neutrophil pRab10 in PSP versus controls was unexpected. The biological relevance of this result is unclear as raised pRab10 levels have consistently been associated with LRRK2‐PD versus controls as a marker of excess kinase activity, which is a disease‐modifying target.^10^, ^11^ Therefore, our result is likely to be a false‐positive finding, noting that in the larger CSF cohort there were no differences in the levels of pRab10 in PSP versus controls. Future replication studies should prioritize more sensitive mass spectrometry methods to quantify a range of LRRK2‐dependent Rab proteins. Of note, a recent analysis of PSP brain tissue lysates and immunohistochemistry revealed that pRab12 labels lysosomal‐like granulovacuolar bodies in neurons and shows co‐pathology with tau inclusions.^18^

Although we did not observe an association between group (PSP vs. controls) and levels of CSF total LRRK2 and pRab10, stratification of results by LRRK2 PD risk SNP (rs76904798) status revealed significantly higher levels of CSF total LRRK2 in risk allele (T) carriers versus CC genotype in the PSP group. This suggests that a subset of PSP patients have genetically determined pathogenically raised LRRK2 levels and this occurs independently of pRab10 such that these patients may benefit from LRRK2‐lowering therapies, including ASOs, as opposed to kinase inhibitors. This conclusion is further reinforced by our finding of baseline monocyte total LRRK2 levels predicting 1‐year change in PSPRS scores in the PSP group. The effect of monocyte LRRK2 levels on disease progression may relate to peripheral immune response‐driven neuroinflammation or be due to a specific effect in monocyte or microglial lineage cells.^19^ Furthermore, the therapeutic potential of targeting LRRK2 in PSP has been shown in a recent whole‐genome CRISPR screen which highlighted the role of LRRK2 in controlling the endocytosis of monomeric tau in human neurons.^20^

Of note, we have previously shown that common variation near the LRRK2 locus (rs2242367) determines survival in PSP, and carrying the alternate allele at rs2242367 was associated with reduced survival and increased expression of LRRK2 in whole blood.^5^ However, in this study we only observed a non‐significant (*P* = 0.08) trend towards rs2242367 genotype status determining CSF total LRRK2 levels in PSP. It should be noted that whilst rs76904798 and rs2242367 are in low *r* ^2^ linkage disequilibrium, they are within 200 kb of each other and have a high D′, suggesting that they may be part of a haplotype block which determines disease progression in PSP. It is also possible that rs76904798 and rs2242367 have differing effects on regulating LRRK2 expression in different cells and/or cellular states that may be relevant to the development of PD and PSP. In line with this, a recent study showed that rs76904798 status determines the level of microglial LRRK2 expression in postmortem brain tissue and in a patient‐induced pluripotent stem cell‐derived microglia model.^21^

We found significantly higher levels of urine BMP species in PSP versus controls. BMPs are part of a broad group of phospholipids that are important for membrane function in the endosome–lysosome compartment; however, the biological relevance of individual BMP isoforms is unknown. In previous studies that have detected higher levels of urine BMP in LRRK2‐PD versus sporadic PD and controls, significant results were obtained for both the total di‐22:6 BMP and total di‐18:1 BMP isoforms.^12^, ^13^ Therefore, our significant result for total di‐22:6 BMP but not total di‐18:1 BMP is likely to be related to statistical power as opposed to an isoform‐specific effect in PSP, noting that there was a non‐significant trend towards higher levels of total di‐18:1 BMP in PSP versus controls. Although raised urine BMP has previously been detected in LRRK2‐PD, it has also been found in lysosomal storage disorders including Niemann–Pick type C disease, such that it is considered a non‐specific marker of lysosomal dysfunction.^22^ However, in PSP, raised urine BMP levels may potentially be driven by LRRK2 dysfunction due to the positive correlation we observed between urine di‐22:6 BMP and CSF LRRK2 levels, although further work is required to explore this hypothesis. In the PPMI cohort, urine BMP levels remained stable over 2 years in LRRK2‐PD participants. Furthermore, in LRRK2‐PD, GBA‐PD, and sporadic PD, baseline urine BMP levels did not predict disease progression as measured by striatal dopamine transporter (DaT) imaging, MDS‐UPDRS‐III, and MoCA. Altogether, these results suggested that urine BMP may be used as a target modulation biomarker in clinical trials for monogenic and sporadic PD, but not as a prognostic or disease progression biomarker.^13^

The main limitation of this study was the small sample size and limited number of PSP participants that had 1‐year clinical follow‐up data available, which is why the results of our progression analyses need to be replicated. Independent replication in larger genetically characterized PSP cohorts alongside sporadic PD and LRRK2‐PD‐positive control groups will be essential to validate our findings. Although WGS data were only available for a subset of our participants, this (a) confirmed that our finding of raised urine BMPs in PSP versus controls was not impacted by the inclusion of PSP cases that are LRRK2 or GBA carriers, although it should be noted that dedicated Gauchian variant calling^23^ was not available for PSP cases and (b) validated the LGC genotyping results that were used to do the pQTL analyses.

In conclusion, biochemically defined lysosomal dysfunction is evident in PSP. We have also shown a pQTL effect of rs76904798 on CSF LRRK2 levels as well as baseline monocyte total LRRK2 levels predicting 1‐year change in PSPRS scores. Altogether, these findings suggest that genetic and biochemical stratification may identify PSP patients that would benefit from LRRK2‐targeting therapies.

## Author Roles

(1) Research Project: A. Conception, B. Design, C. Execution; (2) Statistical Analysis: A. Design, B. Data Analysis, C. Review and Critique; (3) Manuscript Preparation: A. Writing of the First Draft, B. Editing of the Final Version.

L.K.N.: 1C, 2B, 3A, 3B.

E.M.S.: 1B, 3B.

E.J.: 1A, 1B, 1C, 2A, 2B, 2C, 3A, 3B.

All other authors: 1C, 3B.

## Supporting information


**Figure S1.** Representative immunoblots for control and progressive supranuclear palsy (PSP) participants 1 and 2 in monocytes and neutrophils.
**Figure S2**. Progressive supranuclear palsy (PSP) versus control quantitative immunoblotting plots for DMSO‐ and MLi‐2‐treated neutrophil and monocyte samples.
**Figure S3**. Heatmap of Spearman's rho biomarker correlations in the control group.
**Table S1**. List of antibodies used in the immunoblot analysis of neutrophil and monocyte samples.
**Table S2**. Parkinson's disease/parkinsonism genes screened for pathogenic variants using whole‐genome sequencing and NeuroBooster Array data.
**Table S3**. Progressive supranuclear palsy (PSP) versus control comparisons of urine total di‐22:6 and di‐18:1 bis(monoacylglycerol)phosphate (BMP) levels and associated isoforms.
**Table S4**. Blood (neutrophil and monocyte) and cerebrospinal fluid total LRRK2 and pRab10 levels in progressive supranuclear palsy (PSP) and control groups stratified by rs2242367 and rs76904798 genotype status.
**Table S5**. Linear regression models using baseline levels of neutrophil, monocyte, urine, and cerebrospinal fluid measures to predict 1‐year change in the Progressive Supranuclear Palsy Rating Scale (PSPRS) score.
**Table S6**. Linear regression models using baseline levels of neutrophil, monocyte, urine, and cerebrospinal fluid measures to predict 1‐year change in Movement Disorder Society‐Unified Parkinson's Disease Rating Scale‐Part III (MDS‐UPDRS‐III) and Montreal Cognitive Assessment (MoCA) scale scores.

## Data Availability

The raw data used for analyses in this study will be considered for sharing in an anonymized format on request from a qualified investigator to the corresponding authors for the purposes of replicating procedures and results.
