# Supplementary material for: Biomarkers of Leucine‐Rich Repeat Kinase 2 (LRRK2) and Lysosomal Dysfunction in Progressive Supranuclear Palsy
Source: Mov Disord. 2026 Apr 15;41(7):1740–9. doi: 10.1002/mds.70295 (PMC13387967; doi:10.1002/mds.70295)
Supplement: Supplementary file 1 — Figure S1. Representative immunoblots for control and progressive supranuclear palsy (PSP) participants 1 and 2 in monocytes and neutrophils. Figure S2. Progressive supranuclear palsy (PSP) versus control quantitative immunoblotting plots for DMSO‐ and MLi‐2‐treated neutrophil and monocyte samples. Figure S3. Heatmap of Spearman's rho biomarker correlations in the control group. Table S1. List of antibodies used in the immunoblot analysis of neutrophil and monocyte samples. Table S2. Parkinson's disease/parkinsonism genes screened for pathogenic variants using whole‐genome sequencing and NeuroBooster Array data. Table S3. Progressive supranuclear palsy (PSP) versus control comparisons of urine total di‐22:6 and di‐18:1 bis(monoacylglycerol)phosphate (BMP) levels and associated isoforms. Table S4. Blood (neutrophil and monocyte) and cerebrospinal fluid total LRRK2 and pRab10 levels in progressive supranuclear palsy (PSP) and control groups stratified by rs2242367 and rs76904798 genotype status. Table S5. Linear regression models using baseline levels of neutrophil, monocyte, urine, and cerebrospinal fluid measures to predict 1‐year change in the Progressive Supranuclear Palsy Rating Scale (PSPRS) score. Table S6. Linear regression models using baseline levels of neutrophil, monocyte, urine, and cerebrospinal fluid measures to predict 1‐year change in Movement Disorder Society‐Unified Parkinson's Disease Rating Scale‐Part III (MDS‐UPDRS‐III) and Montreal Cognitive Assessment (MoCA) scale scores. [file MDS-41-1740-s001.docx]

**Biomarkers of LRRK2 and lysosomal dysfunction in Progressive Supranuclear Palsy – Supplementary material**

| **Section** | | **Page** |
| --- | --- | --- |
| 1) | Methods: Immunoblotting | 2 |
| 2) | Table 1: List of antibodies used in the immunoblot analysis of neutrophil and monocyte samples | 3 |
| 3) | Table 2: PD/Parkinsonism genes screened for pathogenic variants using WGS and NeuroBooster Array data | 4 |
| 4) | Figure 1: Representative immunoblots for control and PSP participants 1 and 2 in monocytes and neutrophils | 5 |
| 5) | Figure 2: PSP vs. control quantitative immunoblotting plots for DMSO and MLi-2 treated neutrophil and monocyte samples | 6 |
| 6) | Table 3: PSP vs. control comparisons of urine total di-22:6 and di-18:1 BMP levels and associated isoforms | 7 |
| 7) | Table 4: Blood (neutrophil and monocyte) and CSF total LRRK2 and pRab10 levels in PSP and control groups stratified by rs2242367 and rs76904798 genotype status | 8 |
| 8)  9)  10) | Figure 3: Heatmap of Spearman’s rho biomarker correlations in the control group  Table 5: Linear regression models using baseline levels of neutrophil, monocyte, urine and CSF measures to predict 1 year change in the PSP rating scale score  Table 6: Linear regression models using baseline levels of neutrophil, monocyte, urine and CSF measures to predict 1 year change in MDS-UPDRS-III and MoCA scale scores | 9  10  11 |

**Methods: Immunoblotting**

In preparation for multiplexed immunoblotting, the protein concentrations of all cleared neutrophil and monocyte lysates were determined via the BCA assay, and samples were made up to a protein concentration of 2 mg/ml in 4x NuPAGE LDS sample buffer supplemented with 5% (v/v) β-mercaptoethanol. For each sample, 20 μg of protein was loaded in duplicates onto 20-well commercial NuPAGE 4-12% Bis-Tris gels and electrophoresed in MOPS SDS running buffer at 90V for 15 minutes and then 140V until the dye-front was at the bottom of the gel. The electrophoresed proteins were transferred from the gels onto nitrocellulose membranes using wet transfer at 90V for 90 minutes in transfer buffer (48 mM Tris, 39 mM glycine, 20% (v/v) methanol). Following transfer, the membranes were stained with Ponceau S and cut to separate the proteins of interest (LRRK2, Rab10, and the housekeeping protein, GAPDH). The ponceau strain was washed off with TBS-T (20 mM Tris, 150 mM NaCl, 0.2% (v/v) Tween20), after which the membranes were blocked at room-temperature for 1 hour in 5% (w/v) milk in TBS-T. Following washing in TBS-T, the membranes were incubated overnight at 4 °C in primary antibodies (multiplexed total LRRK2 and pSer935 LRRK2, multiplexed total Rab10 and pThr73 Rab10, and GAPDH (see Supplementary Table 1)) diluted in a TBS-T buffer with 5% (w/v) BSA and 0.02% sodium azide. From this point forward, the membranes were kept in the dark. The next day, the membranes were washed 3x5 minutes in TBS-T and incubated in secondary antibodies (goat anti-rabbit IRDye 800CW and/or goat anti-mouse IRDye 680RD (see Supplementary Table 1)) diluted 1:10000 in TBS-T at room temperature for 1 hour. The membranes were washed 3x10 minutes in TBS-T and imaged on a LI-COR Odyssey CLx scanner. Finally, the signal intensities were quantified in ImageStudio.

**Table 1**

| **Primary antibodies** | | | | |
| --- | --- | --- | --- | --- |
| **Antibody** | **Species** | **Catalogue no** | **Dilution** | **Final concentration** |
| Total LRRK2 | Mouse | NeuroMab #75-253 | 1:1000 | 1 µg/mL |
| pSer935 LRRK2 | Rabbit | Abcam #ab133450 | 1:1000 | 1 µg/mL |
| Total Rab10 | Mouse | Nanotools #0680-100 | 1:500 | 1 µg/mL |
| pThr73 Rab10 | Rabbit | Abcam #ab230261 | 1:1000 | 1 µg/mL |
| GAPDH | Mouse | Santa Cruz #sc-32233 | 1:10000 | 0.01 µg/mL |
| **Secondary antibodies** | | | | |
| anti-mouse IRDye 680RD | Goat | LI-COR #926-68070 | 1:10000 | 0.1 µg/mL |
| anti-rabbit IRDye 800CW | Goat | LI-COR #926-32211 | 1:10000 | 0.1 µg/mL |

**Table 1:** List of antibodies used in the immunoblot analysis of neutrophil and monocyte samples.

**Table 2**

| ATP13A2 | LYST | SLC30A10 |
| --- | --- | --- |
| ATP1A3 | MAPT | SLC39A14 |
| C19orf12 | OPA3 | SLC6A3 |
| CSF1R | PANK2 | SNCA |
| DCTN1 | PARK7 | SPG11 |
| DNAJC6 | PDGFB | SPR |
| FBXO7 | PINK1 | SYNJ1 |
| FTL | PLA2G6 | TH |
| GBA | PRKN | TUBB4A |
| GCH1 | PRKRA | VPS13A |
| GRN | PTRHD1 | VPS35 |
| LRRK2 | RAB39B | WDR45 |

**Table 2:** PD/Parkinsonism genes screened for pathogenic variants using WGS and NeuroBooster Array data.

**Figure 1**

**
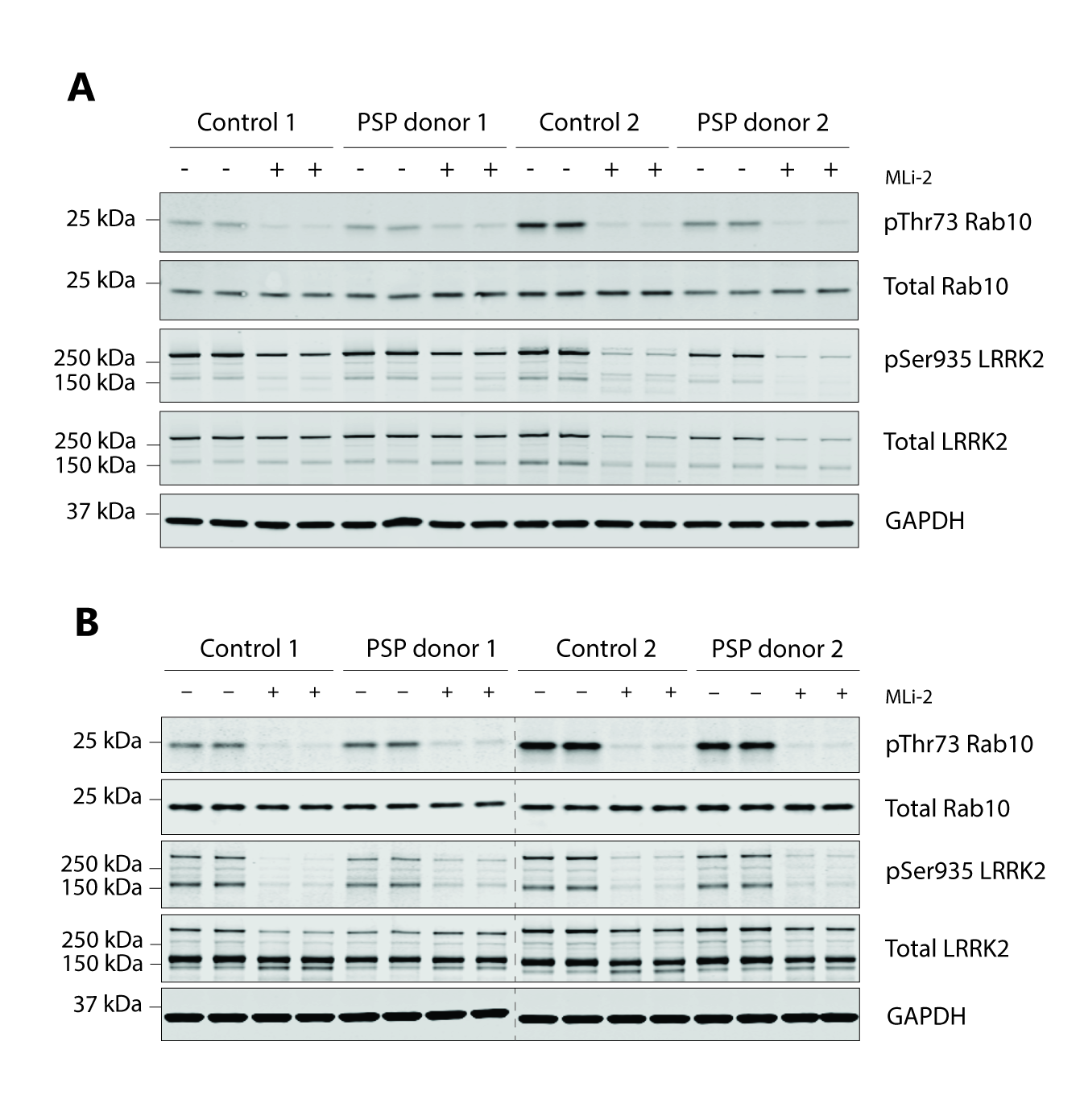
**

**Figure 1:** Representative immunoblots for control and PSP participants 1 and 2 in: a) monocytes; b) neutrophils. All samples were run in duplicates with 20 μg of lysate being loaded into each well. The housekeeping protein, GAPDH (glyceraldehyde-3-phosphate dehydrogenase), was used as a loading control and to normalize the quantified signal intensities of the total protein bands for LRRK2 and Rab10.

**Figure 2**

**Figure 2:** PSP vs. control quantitative immunoblotting plots for DMSO and MLi-2 treated samples regarding: a) neutrophil phosphorylated LRRK2 levels normalised by total LRRK2; b) neutrophil total LRRK2 levels normalised by GAPDH; c) neutrophil phosphorylated Rab10 levels normalised by total Rab10; d) neutrophil total Rab10 levels normalised by GAPDH; e) monocyte phosphorylated LRRK2 levels normalised by total LRRK2; f) monocyte total LRRK2 levels normalised by GAPDH; g) monocyte phosphorylated Rab10 levels normalised by total Rab10; h) monocyte total Rab10 levels normalised by GAPDH. Group comparisons were done using logistic regression that adjusted for sex and age at testing.

**Table 3**

| **Biomarker** | **OR** | **95% CI** | **p-value** |
| --- | --- | --- | --- |
| Total di-18:1-BMP | 1.06 | (0.94, 1.19) | 0.33 |
| di.18.1.BMP.2.2 | 1.02 | (0.89, 1.18) | 0.74 |
| di.18.1.BMP.2.3 | 1.79 | (0.86, 3.70) | 0.12 |
| di.18.1.BMP.3.3 | 16.30 | (2.01, 131.00) | **0.01** |
| Total.di.22.6.BMP | 1.04 | (1.00, 1.08) | **0.04** |
| di.22.6.BMP.2.2 | 1.04 | (0.99, 1.10) | 0.10 |
| di.22.6.BMP.2.3 | 1.14 | (1.00, 1.30) | 0.05 |
| di.22.6.BMP.3.3 | 1.35 | (0.98, 1.84) | 0.06 |

**Table 3:** PSP vs. control comparisons of urine total di-22:6 and di-18:1 BMP levels and their associated isoforms. Group comparisons were done using logistic regression that adjusted for sex and age at testing.

**Table 4**

| **SNP: rs2242367** | | | | |
| --- | --- | --- | --- | --- |
| **Biomarker** | **Group** | **Estimate** | **95% CI** | **p-value** |
| CSF LRRK2 | PSP | 3.21 | (-0.24, 6.67) | 0.08 |
| CSF pRab10 | PSP | -0.08 | (-0.31, 0.14) | 0.47 |
| Neutrophil tLRRK2/GAPDH | PSP | 0.70 | (-0.62, 2.01) | 0.32 |
|  | **Control** | **0.62** | **(0.08, 1.16)** | **0.04** |
| Neutrophil pRab10/tRab10 | PSP | 0.05 | (-0.14, 0.23) | 0.63 |
|  | Control | 0.05 | (-0.13, 0.22) | 0.59 |
| Monocyte tLRRK2/GAPDH | PSP | -0.09 | (-0.46, 0.28) | 0.65 |
|  | Control | -0.23 | (-0.60, 0.14) | 0.24 |
| Monocyte pRab10/tRab10 | PSP | -0.01 | (-0.37, 0.36) | 0.98 |
|  | Control | 0.14 | (-0.28, 0.56) | 0.52 |

| **SNP: rs76904798** | | | | |
| --- | --- | --- | --- | --- |
| **Biomarker** | **Group** | **Estimate** | **95% CI** | **p-value** |
| CSF LRRK2 | **PSP** | **4.45** | **(0.98, 7.92)** | **0.02** |
| CSF pRab10 | PSP | 0.07 | (-0.17, 0.30) | 0.57 |
| Neutrophil tLRRK2/GAPDH | PSP | -0.32 | (-1.56, 0.91) | 0.62 |
|  | Control | -0.33 | (-1.13, 0.48) | 0.44 |
| Neutrophil pRab10/tRab10 | PSP | -0.08 | (-0.25, 0.09) | 0.36 |
|  | Control | -0.10 | (-0.34, 0.14) | 0.41 |
| Monocyte tLRRK2/GAPDH | PSP | -0.04 | (-0.40, 0.33) | 0.85 |
|  | Control | 0.09 | (-0.43, 0.62) | 0.73 |
| Monocyte pRab10/tRab10 | PSP | -0.02 | (-0.38, 0.33) | 0.89 |
|  | Control | -0.35 | (-0.91, 0.21) | 0.24 |

**Table 4:** Blood (neutrophil and monocyte) and CSF total LRRK2 and pRab10 levels in PSP and control groups stratified by rs2242367 and rs76904798 genotype status. Genotype group comparisons were done using linear regression that adjusted for sex, age and disease duration at testing (control group comparisons adjusted for sex and age at testing) with GG (rs2242367) and CC (rs76904798) as reference groups. CI = confidence interval of the estimate. CSF LRRK2 levels in the control group were not included due to the low number of observations.

**Figure 3**

**Figure 3:** a) Heatmap of Spearman’s rho biomarker correlations in the control group with r values highlighted. Circle represents correlation that was also significant in the linear regression analysis; b) neutrophil pRab10 vs. monocyte pRab10 linear plot. A linear regression analysis that adjusted for sex and age at testing was used to generate the p-value.

**Table 5**

| **Clinical scale** | **Biomarker** | **Estimate** | **95% CI** | **p-value** |
| --- | --- | --- | --- | --- |
| PSPRS  (1 year change) | Neutrophil tLRRK2/GAPDH | -6.61 | (-11.40, -1.78) | 0.03 |
|  | Neutrophil pRab10/tRab10 | 3.13 | (-25.90, 32.10) | 0.84 |
|  | **Monocyte tLRRK2/GAPDH** | **17.80** | **(8.38, 27.30)** | **0.008** |
|  | Monocyte pRab10/tRab10 | 0.59 | (-11.90, 13.10) | 0.93 |
|  | CSF LRRK2 | 0.54 | (-0.25, 1.33) | 0.19 |
|  | CSF pRab10 | 8.51 | (-3.07, 20.10) | 0.16 |
|  | Total di-18:1-BMP | -0.19 | (-0.81, 0.44) | 0.58 |
|  | Total di-22:6-BMP | 0.12 | (-0.21, 0.45) | 0.50 |

**Table 5:** Linear regression models using baseline levels of neutrophil, monocyte, urine and CSF measures to predict 1 year change in the PSP rating scale score, adjusted for sex, age and disease duration at baseline testing. Confidence interval = CI, PSP rating scale = PSPRS. Benjamini-Hochberg corrected p-value significance threshold < 0.009.

**Table 6**

| **Clinical scale** | **Biomarker** | **Estimate** | **95% CI** | **p-value** |
| --- | --- | --- | --- | --- |
| MDS - UPDRS-III  (1 year change) | Neutrophil tLRRK2/GAPDH | 0.07 | (-2.42, 2.56) | 0.96 |
|  | Neutrophil pRab10/tRab10 | 9.22 | (-11.50, 29.97) | 0.41 |
|  | Monocyte tLRRK2/GAPDH | 13.30 | (-0.42, 26.97) | 0.09 |
|  | Monocyte pRab10/tRab10 | 4.90 | (-5.11, 14.90) | 0.37 |
|  | CSF LRRK2 | 0.11 | (-0.69, 0.91) | 0.79 |
|  | CSF pRab10 | 5.72 | (-7.45, 18.90) | 0.40 |
|  | Total di-18-1-BMP | 0.17 | (-0.23, 0.58) | 0.42 |
|  | Total di-22-6-BMP | 0.12 | (0.01, 0.23) | 0.06 |
| MoCA    (1 year change) | Neutrophil tLRRK2/GAPDH | 0.34 | (-1.66, 2.34) | 0.74 |
|  | Neutrophil pRab10/tRab10 | -2.51 | (-17.10, 12.10) | 0.74 |
|  | Monocyte tLRRK2/GAPDH | -2.60 | (-12.30, 7.08) | 0.61 |
|  | Monocyte pRab10/tRab10 | -2.03 | (-9.13, 5.06) | 0.58 |
|  | CSF LRRK2 | 0.05 | (-0.26, 0.36) | 0.76 |
|  | CSF pRab10 | 1.48 | (-3.67, 6.62) | 0.59 |
|  | Total di-18-1-BMP | 0.18 | (-0.11, 0.47) | 0.24 |
|  | Total di-22-6-BMP | 0.03 | (-0.08, 0.13) | 0.63 |

**Table 6:** Linear regression models using baseline levels of neutrophil, monocyte, urine and CSF measures to predict 1 year change in MDS-UPDRS-III and MoCA scale scores, adjusted for sex, age and disease duration at baseline testing. Confidence interval = CI, Movement Disorder Society-Unified Parkinson’s Disease Rating Scale part III = MDS-UPDRS III, Montreal Cognitive Assessment = MoCA.
